# Emergency Medicine at the Frontline of Climate Change: The Role of Geographic Information Systems

**DOI:** 10.5811/westjem.47035

**Published:** 2025-07-18

**Authors:** Tushara Surapaneni, Anna Patrikakou, Antigoni Faka, Liz Grant, Andrew Ulrich, Dimitrios Tsiftsis, Eleanor Reid

**Affiliations:** * Yale School of Medicine, Department of Emergency Medicine, New Haven, Connecticut; † 2nd Regional Health Authority of Piraeus and the Aegean islands – Piraeus, Greece; ‡ Harokopio University of Athens, Department of Geography, Athens, Greece; § University of Edinburgh, Global Health Academy, Usher Institute, Edinburgh, United Kingdom; || Nikaia General Hospital, Department of Emergency Medicine, Nikaia, Greece

## INTRODUCTION

The practice of emergency medicine (EM) is becoming inseparable from public health threats like climate change. As leaders in mass casualty management and prehospital care,^1^ emergency clinicians serve at the frontlines of disaster preparedness and response. With the rising incidence of natural disasters causing humanitarian emergencies,^2^ it is imperative for population-based research to incorporate a spatial lens—one that extends beyond hospital departments and fosters collaboration across scientific disciplines. While several medical journals have published articles about the implications of climate change on human health,^3^–^9^ these discussions need to be accompanied by actionable strategies to strengthen disaster preparedness.

Just as meteorologists study weather patterns, EM researchers are uniquely positioned to leverage spatial technology to anticipate disaster-related surges in patient volume and pathology. In recognition that some countries are dually burdened by high climate vulnerability and underdeveloped emergency care systems, we propose geographic information systems (GIS) as an underutilized tool in EM research to strengthen climate resilience. To emphasize the potential of GIS as a powerful supplement to EM research and disaster preparedness planning, particularly in countries with high climate vulnerability, we introduce GIS followed by a case study of Greece to demonstrate its potential.

### What are geographic information systems?

**Figure 1:**
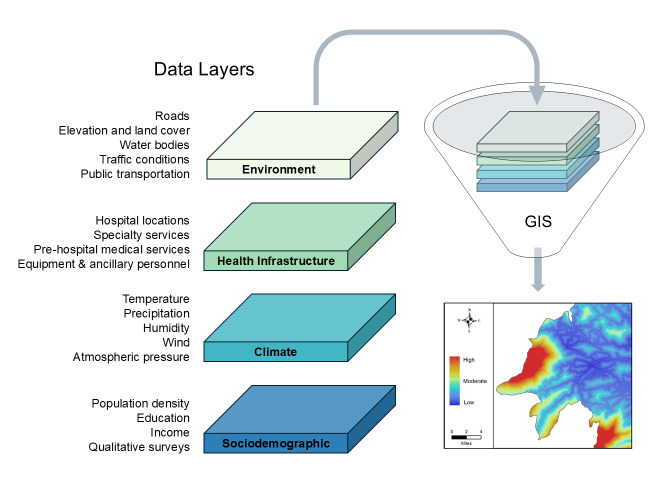
Various data layers can be combined in geographic information systems software to visualize climate hazard risk of a study area.

Key components of disaster management include planning evacuation routes, identifying shelters and medical facilities, mapping disaster risk, developing early warning systems, and monitoring hazard progression.^10^ Creating a climate-resilient disaster preparedness system inherently requires large quantities of geographic data, including elevation, land cover, meteorological observations, and population demographics. Geographic information systems can readily integrate and analyze multiple types of data to create visually informative maps of hazard risk (Figure 1). Several GIS platforms are available, some of which are open access. While they may differ in package offerings, every GIS is a fundamentally iterative system, an essential feature for preparedness systems in the face of a climate crisis with no end date.

### GIS applications for disaster preparedness

As the frequency of natural disasters continues to rise annually, emergency care systems globally can leverage GIS to become proactive, rather than reactive, to the next climate disaster. Analysis is possible at a range of granularity, from census tracts to entire continents, making GIS highly scalable for study areas. The creation of a GIS-based prediction system is accomplished in three phases: data preparation; spatial modeling; and cross-validation.^11^ Historical data from prior natural disasters can be used to test and cross-validate prediction models, enhancing their accuracy. There is a robust body of literature describing methods for prediction modeling of various climate hazards including wildfires,^12^–^13^ landslides,^14^ and floods.^15^–^16^ In the aftermath of a severe weather event, remote-sensing and satellite imagery can be integrated with on-the-ground needs assessments to improve real-time situational awareness. A centralized GIS database also supports continuous data collection from field surveys, minimizing “false perceptions” of ground conditions that often arise during crises.^17^

### Impact of climate change on emergency care systems

Severe weather events such as heatwaves, wildfires, hurricanes, floods, earthquakes, landslides, drought, and tsunamis affect high-, middle- and low-income countries alike, but their impact is felt inequitably. The World Health Organization states:

In the short- to medium-term, the health impacts of climate change will be determined mainly by the vulnerability of populations, their resilience to the current rate of climate change and the extent and pace of adaptation.^18^

**Figure 2:**
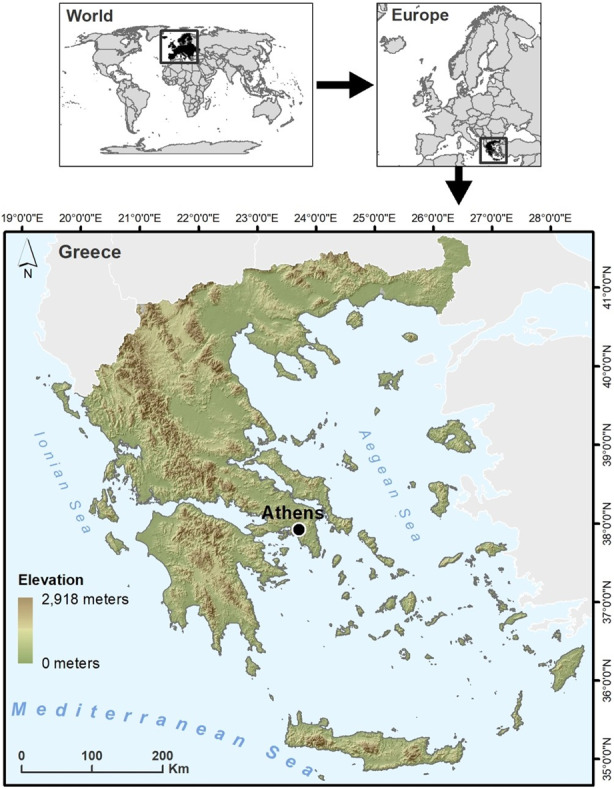
Greece’s geography increases its risk of severe weather events.

Accordingly, it is important to examine cases where natural disasters disproportionately threaten health systems to better understand regional differences in “vulnerability.” Countries with fragile emergency care systems are more susceptible to the long-term impacts of severe weather events due to a lack of resources to appropriately prepare and respond. Many also face geographical constraints to service delivery in remote communities, such as underdeveloped road networks, challenging terrain, and island chains. Even during non-disaster times, the availability of specialized equipment and EM-trained staff varies across countries. Greece (Figure 2) provides an example of a country coping with challenging geography, an underdeveloped emergency care system, and high frequency of severe weather events.

### Case study: Greece’s climate vulnerabilities and emergency care challenges

We present Greece as a case study of a country with high vulnerability to climate stressors given its geography, extreme heat, and surrounding seismic activity. Its mountainous terrain creates localized weather conditions, leading to flash floods in some regions and droughts in others. Earthquakes, which have occurred as recently as February 2025,^19^ are common due to Greece’s location on tectonic plate boundaries and can lead to landslides and infrastructure damage. The Mediterranean climate contributes to severe wildfires every summer, while Greece’s extensive coastline and islands are exposed to flooding from cyclones and hurricanes. Climate change intensifies these threats, as increasing temperatures and rising sea levels lead to more frequent heatwaves and storms, coastal erosion, ecosystems disturbance and, consequently, significant public health threats. From 1980 to 2020, Greece experienced an average of 26 floods per year. Between 2014–2018, floods and earthquakes were the most frequent natural disasters, but wildfires and heatwaves became increasingly prevalent from 2018 to 2023.^20^–^21^ In a list of 35 European nations, Greece ranked sixth highest in the number of heat-related deaths during the summer of 2022.^22^ Climate predictions for Greece have estimated that the number of days with extreme fire risk will increase by 10–15 days annually, with upwards of 15–20 heatwaves per year by 2050, along with increased flash flood events.^23^–^25^

As extreme weather events rise, so will the number of climate-related health conditions, injuries, and deaths. The growing health risks associated with climate change represent an imminent threat to the health of entire populations, and the emergency department (ED) plays a critical role as the first point of contact for people seeking urgent medical care. In Greece, EM is a developing specialty, with a limited number of EM-trained physicians and persistent staffing shortages in public hospitals. The centralized oversight of the Greek Ministry of Health can lead to delayed responses to critical staffing needs, particularly during seasonal influxes of refugees and tourists. On Greece’s islands, baseline challenges of understaffing, a lack of EM-trained attendings, and a fluctuating census of non-permanent residents are further complicated by the hospitals’ remote locations, making them reliant on air ambulances for patient transfers to the mainland.^26^

### GIS applications for disaster preparedness in Greece

In Greece, GIS has the potential to play a vital role in improving climate resilience and minimizing health threats from severe weather events. Greek researchers already use GIS to map fire-prone areas, modeling temperature, humidity, wind speed, and land cover data.^27^–^29^ These spatial prediction models enable better resource allocation, evacuation planning, and strategic placement of firebreaks. Remote sensing and satellite data have also been incorporated for real-time monitoring, providing acute data on fire location, size, and direction to aid emergency response efforts.^30^ To assess flood risk, several studies in Greece have used topographic and rainfall data to design flood protection projects.^31^–^33^ The next step is to integrate climate research with sociodemographic and health data to create risk maps of conditions such as heat-related illnesses, pollution-related respiratory diseases, and traumatic injuries after natural disasters. Future research studies can use GIS to do the following:

- Predict the health impacts of high temperatures, wildfires, and floods on vulnerable groups of patients;
- Guide hospital administration to preemptively request additional staff and resources, enabling timely infrastructure improvements and coordinated response effort;
- Inform equitable distribution of resources to underserved or geographically vulnerable communities.

## CONCLUSION

Geography is a key determinant of health, but it is often overlooked in emergency medicine research. Amid the shift-to-shift demands of emergency medicine, it can be difficult to contextualize how environmental changes are affecting the health of communities. We argue that disaster preparedness systems in climate-vulnerable countries could be more effective if specialists in medicine, climate science, geography, and epidemiology collaborated on data-driven solutions. Emergency medicine researchers can partner with geospatial experts within their hospitals or local universities to harness the potential of geographic information systems. By forecasting *“when”* and *“where”* climate-related health conditions will escalate, GIS can empower health systems to prepare for *“who”* arrives at the emergency department.
